# Anti‐*Helicobacter pylori* therapy in localized gastric mucosa‐associated lymphoid tissue lymphoma: A prospective, nationwide, multicenter study in Japan

**DOI:** 10.1111/hel.12474

**Published:** 2018-03-04

**Authors:** Katsuya Sugizaki, Akira Tari, Yasuhiko Kitadai, Ichiro Oda, Shotaro Nakamura, Tadashi Yoshino, Toshiro Sugiyama

**Affiliations:** ^1^ Medical HQs Eisai Co., Ltd. Tokyo Japan; ^2^ Division of Gastroenterology Department of Internal Medicine Hiroshima Red Cross Hospital & Atomic‐bomb Survivors Hospital Hiroshima Japan; ^3^ Department of Health Sciences Faculty of Human Culture and Science Prefectural University of Hiroshima Hiroshima Japan; ^4^ Endoscopy Division National Cancer Center Hospital Tokyo Japan; ^5^ Division of Gastroenterology Department of Internal Medicine School of Medicine Iwate Medical University Morioka Japan; ^6^ Department of Pathology Okayama University Graduate School of Medicine Dentistry and Pharmaceutical Sciences Okayama Japan; ^7^ Department of Gastroenterology and Hematology Graduate School of Medicine and Pharmaceutical Sciences University of Toyama Toyama Japan

**Keywords:** gastric MALT lymphoma, *Helicobacter pylori* eradication, prospective nationwide multicenter study, rabeprazole‐based triple therapy

## Abstract

**Background:**

*Helicobacter pylori* eradication therapy was approved in Japan for the first‐line, standard treatment of *H. pylori*‐positive gastric mucosa‐associated lymphoid tissue (MALT) lymphoma. Although several retrospective studies or small‐scale single‐center studies have been reported, a prospective, large‐scale, nationwide, multicenter study has not been reported from Japan.

**Materials and Methods:**

We conducted a prospective, nationwide, multicenter study to evaluate the clinical efficacy of rabeprazole‐based triple *H. pylori* eradication therapy for patients with localized gastric MALT lymphoma in practice‐based clinical trial. A total of 108 *H. pylori*‐positive patients with stage I/II_1_ gastric MALT lymphoma underwent *H. pylori* eradication therapy. The primary endpoints were complete remission (CR) rate and the rate of transfer to secondary treatment. The secondary endpoints were CR maintenance duration and overall survival (OS).

**Results:**

CR of lymphoma was achieved in 84 of 97 patients (86.6%), during the period 2.0‐44.7 months (median, 5.3 months) after starting *H. pylori* eradication treatment. CR was maintained in 77 of 81 patients (95.1%) for 0.4‐53.2 months (median, 33.1 months). Secondary treatments (radiotherapy, rituximab, or gastrectomy) for gastric MALT lymphoma were needed in 10 of the 97 patients (10.31%). During follow‐up, OS rate was 96.9% (94/97) and the causes of 3 deaths were not related to lymphoma.

**Conclusions:**

Rabeprazole‐based *H. pylori* eradication therapy demonstrated a high CR rate, long CR maintenance, and a good OS for patients with localized gastric MALT lymphoma in this prospective, practice‐based, multicenter study.

## 1 INTRODUCTION

Extranodal marginal zone B‐cell lymphoma of mucosa‐associated lymphoid tissue (MALT lymphoma) is a low‐grade lymphoma arising from various extranodal organs, such as the digestive tract, thyroid, lung, salivary gland, ocular adnexa, liver, skin, and breast.^1^ This pathology was first proposed as a distinct disease entity by Isaacson and Wright in 1983,^2^ and is considered to be triggered by persistent chronic inflammation.^1^, ^2^ Gastric MALT lymphoma is an indolent lymphoma, and *Helicobacter pylori* eradication induces clinical and histological regression of the disease in the majority of cases, as first reported by Wotherspoon et al.^3^ Approximately 60%‐80% of *H. pylori*‐positive gastric MALT lymphomas achieve complete histological response after *H. pylori* eradication.^3^, ^4^, ^5^ *H. pylori* eradication therapy is currently recommended as the first‐line treatment for all patients with gastric MALT lymphoma, as described in guidelines from the United States, Europe, and Japan.^6^, ^7^, ^8^, ^9^, ^10^, ^11^

In Japan, *H. pylori* eradication therapy was approved for the treatment of *H. pylori*‐positive gastric MALT lymphoma in 2010, based on single‐center retrospective studies.^12^, ^13^ To date, however, no well‐controlled, prospective, multicenter studies have been reported from Japan. We therefore conducted a prospective, nationwide, multicenter study to confirm the efficacy and safety of this treatment in Japan.

## 2 MATERIALS AND METHODS

### 2.1 Subjects and study design

This prospective, multicenter trial (ClinicalTrials.gov, NCT01264822) was conducted at 34 hospitals in Japan from December 2010 to February 2016. Subjects were patients with *H. pylori*‐positive gastric MALT lymphoma in stage I or II_1_, as determined by the Lugano staging system.^14^ The diagnosis of MALT lymphoma was based on the histopathological criteria according to the World Health Organization classification,^1^ compatible with grade 4 or 5 in the Wotherspoon's histological score.^3^ Patients were excluded if they showed diffuse large B‐cell lymphoma (DLBCL) or had previously received any other oncological treatment for gastric MALT lymphoma. Candidate patients were registered by physicians in each hospital within 5 days after the start of *H. pylori* eradication therapy.

*H. pylori* status was determined according to histology, culture, rapid urease test, ^13^C urea breath test (UBT), *H. pylori* stool antigen test (HpSA), and/or serology. *H. pylori* infection was judged as positive if at least one of the tests yielded a positive result, and as negative when all tests were negative. The endoscopic type of MALT lymphoma was classified as superficial, ulcerative, elevated, or other.^15^ Endoscopic ultrasound (EUS) was performed to evaluate the depth of tumor invasion and degree of perigastric lymphadenopathy.^15^ The status of t(11;18)(q21;q21)/*API2‐MALT1* was investigated by reverse‐transcription polymerase chain reaction and/or fluorescence in situ hybridization (FISH).^15^

All patients underwent *H. pylori* eradication with rabeprazole (RPZ)‐based triple therapy (RPZ 10 mg + amoxicillin 750 mg + clarithromycin [CAM] 200 or 400 mg, or metronidazole [MNZ] 250 mg) twice a day for 7 days, as a regimen approved by the Japanese governmental healthcare system. Successful eradication was basically evaluated using the UBT or HpSA according to the Maastricht IV consensus.^10^ After successful eradication, the follow‐up observation was started from the first day of successful *H. pylori* eradication, and the related parameters including histopathology and transition to secondary treatment for MALT lymphoma were evaluated. The follow‐up period after eradication therapy in each patient was basically set to ≥24 months.

This study was designed as a prospective, practice‐based, observation study and conducted in compliance with Good Post‐marketing Study Practice (GPSP), a ministerial ordinance of the Ministry of Health, Labour and Welfare of Japan. Patient consent was therefore not sought, but the right to opt out was explained by survey physicians according to the Japanese Ethical Rule for clinical observation studies. All the data collection and analyses were performed at Eisai Co., Ltd. (Tokyo Japan).

### 2.2 Histological evaluation

Histological diagnosis of MALT lymphoma was performed from biopsy specimens by pathologists in each participating hospital. When a definitive diagnosis of MALT lymphoma could not be confirmed in the hospital, the central pathologist (T.Y.) reviewed the cases. Biopsy specimens after *H. pylori* eradication were also evaluated by pathologists in each the hospital, while relapsed cases and suspicious cases were reviewed by the central pathologist (T.Y.). Histopathological evaluation after treatment was carried out using the Groupe d'Etude des Lymphomes de l'Adulte (GELA) histological grading system, with classification as either complete histological response (ChR), probable minimal residual disease (pMRD), responding residual disease (rRD) or no change (NC)^8^, ^16^ or Wotherspoon's histological score (0‐5).^3^ Complete remission (CR) of lymphoma was defined as ChR or pMRD in the GELA system, or grades 0 or 1 in Wotherspoon's score, with non‐CR defined as any other category. Histopathological examinations were performed every 3 months until 1 year after successful eradication, and every 6 months thereafter. Treatment failure was defined as relapse after CR or progressive disease (PD) including transformation into DLBCL, or non‐CR after successful *H. pylori* eradication.

### 2.3 Secondary treatment for patients with treatment failure

When a lymphoma was judged by a physician as not responsive to eradication treatment, the patients underwent other oncological treatments (radiotherapy, chemotherapy, rituximab, or surgical resection). They were defined as in transition to secondary treatment. The timing of transfer to secondary treatment was judged by the physician, because this trial was not an interventional study, but an observation study, as defined in the Japanese Ethical Rule for clinical observation studies.

### 2.4 Endpoints and statistical analysis

Primary endpoints were CR rate by *H. pylori* eradication therapy alone and the rate of transfer to secondary treatment. CR rate was calculated as the proportion of patients in the efficacy analysis set who responded to *H. pylori* eradication therapy alone (ChR or pMRD in the GELA system, or Wotherspoon score 0 or 1). In this analysis, patients who achieved CR after secondary treatment were not regarded as achieving CR by *H. pylori* eradication therapy. Secondary endpoints were the rate of CR maintenance and overall survival (OS), as well as rates of successful *H. pylori* eradication and adverse reactions to RPZ‐based triple therapy. The 95% confidence intervals (CIs) were calculated with F‐distribution. Subgroup analyses were performed for response rate and background factors by Fisher's exact probability test or the chi‐square test, with a two‐tailed significance level of 5%. Probabilities of CR maintenance, secondary treatment transfer, and OS were analyzed by the Kaplan‐Meier method.

## 3 RESULTS

### 3.1 Clinical features of patients

Among the 108 registered patients, one was excluded for a registration violation and 107 were included in the safety analysis set. Among the 107 patients in the safety analysis set, 10 patients were excluded for either a lack of efficacy evaluation (n = 4), using a regimen unapproved in Japan (n = 4), or lacking confirmed histological evidence of MALT lymphoma (n = 2). The remaining 97 patients were included in the efficacy analysis set.

As shown in Table 1, median age was 65 years (range, 35‐85 years), and 50 patients (52%) were women. As for clinical stage, 95 patients (98%) had stage I disease. Wotherspoon histological score was grade 5 in 65 patients (67%). Endoscopic findings showed superficial type in 89 patients (92%). Depth of lymphoma invasion was evaluated using EUS in 37 patients (38%), of whom 27 (73%) were diagnosed as having intramucosal tumors. The t(11;18)(q21;q21)/*API2‐MALT1* translocation was successfully investigated in 73 patients (75%), of whom only 1 patient showed positive results for the translocation.

**Table 1:** The clinical characteristics and CR rate by *Helicobacter pylori* eradication in 97 patients

Characteristics	N (%)	No. of CR/all patients	CR rate, % (95% CI)	*P* value
Age (years)				
Median (range)	65 (35‐85)			
64≥	46 (47)	37/46	80 (66.1‐95.2)	0.135
65≤	51 (53)	47/51	92 (81.1‐87.8)	
Sex				
Male	47 (48)	41/47	87 (74.3‐95.2)	1.000
Female	50 (52)	43/50	86 (73.3‐94.2)	
Clinical stage				
I	95 (98)	83/95	87 (78.9‐93.3)	0.251
II_1_	2 (2)	1/2	50 (1.3‐98.7)	
Histological score^a^				
Grade 4	32 (33)	26/32	81 (63.6‐92.8)	0.345
Grade 5	65 (67)	58/65	89 (79.1‐95.6)	
Dominant site of lesion^a^				
Proximal third (U)	40 (41)	34/40	85 (70.2‐94.3)	NE
Middle third (M)	76 (78)	65/76	86 (75.6‐92.6)	
Distal third (L)	20 (21)	17/20	85 (62.1‐96.8)	
Endoscopic type				
Superficial	89 (92)	79/89	89 (80.3‐94.5)	0.016
Ulcerative	7 (7)	5/7	71 (29.0‐96.3)	
Elevated	1 (1)	0/1	0 (0.0‐97.5)	
Depth by EUS				
Mucosa	27 (28)	24/27	89 (70.8‐97.7)	0.597
Submucosa or deeper	10 (10)	8/10	80 (44.4‐97.5)	
t(11;18)/*API2‐MALT1* (n = 73)				
Positive	1 (1)	1/1	100 (2.5‐100.0)	1.000
Negative	72 (99)	63/72	88 (77.6‐94.1)	

CR, complete remission; NE, not evaluated, CI, confidence interval.

Wotherspoon's score.

a Major site.

### 3.2 Successful *Helicobacter pylori* eradication

Successful *H. pylori* eradication was achieved in 86 of 97 patients (88.7%). The eradication rate was 87.8% (79/90) with CAM‐based therapy and 100.0% (7/7) with MNZ‐based therapy. All patients with failure of CAM‐based therapy were treated using MNZ‐based therapy. UBT (n = 87), HpSA (n = 1), culture (n = 6), and histology (n = 3) were used to identify successful eradication. Median time to successful eradication after completion of eradication therapy was 6.9 weeks (range, 4.0‐24.7 weeks).

### 3.3 Response to *Helicobacter pylori* eradication therapy

**Figure 1:**
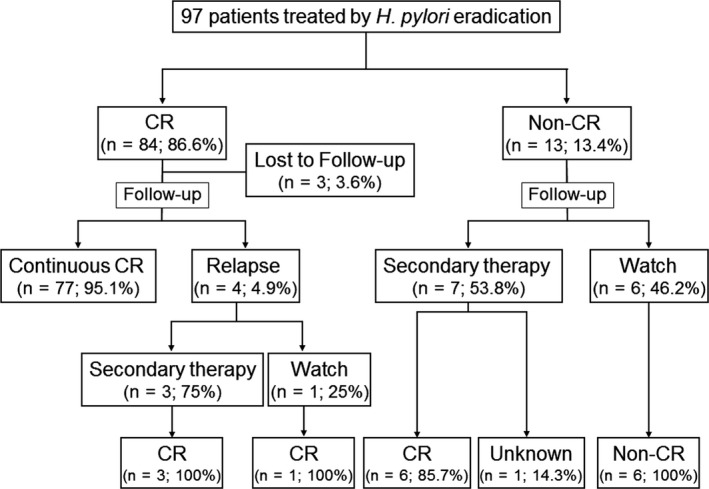
Clinical courses of the 97 study patients. CR, complete remission

Clinical course and outcomes in the efficacy analysis set of 97 patients are shown in Figure 1. CR was achieved in 86.6% of patients (84/97) using *H. pylori* eradication treatment alone. The median interval to CR after the start of the successful eradication treatment was 5.3 months (range, 2.0‐44.7 months), and 61.9% of CR patients (52/84) achieved CR within 6 months after starting successful eradication treatment.

The clinical characteristics and CR rates in the 97 patients are shown in Table 1. Cases with superficial type on endoscopy showed a significantly higher CR rate than cases with other type (*P *=* *.016). As t(11;18)(q21;q21)/*API2‐MALT1* translocation was detected in only 1 case, no significant difference was identifiable, probably due to patient enrollment bias.

### 3.4 Relapse and duration of complete remission

**Figure 2:**
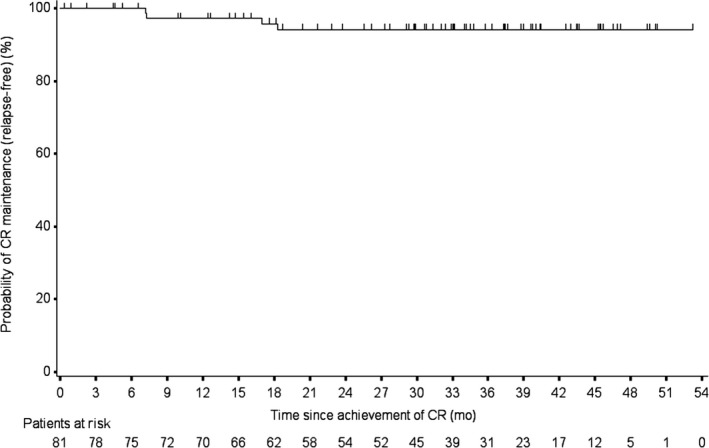
Kaplan‐Meier estimates of complete remission (CR) maintenance (relapse‐free) after achievement of CR

During follow‐up, relapse of MALT lymphoma was observed in 4 of 81 patients (4.9%). In the remaining 77 patients (95.1%) achieved CR by *H. pylori* eradication therapy, CR was maintained until the end of the observation period without any additional treatment. Median duration of CR maintenance was 33.1 months (range, 0.4‐53.2 months). Kaplan‐Meier estimates for the cumulative probability of CR maintenance were 97.3% (95%CI, 89.6%‐99.3%) at 12 months and 94.2% (95%CI, 85.3%‐97.8%) at each of 24 months, 36 months and 48 months (Figure 2). In 54 of the 81 patients (66.7%) achieved CR, the remission has been maintained for more than 2 years. The one patient with t(11;18)(q21;q21)/*API2‐MALT1* translocation achieved CR after successful *H. pylori* eradication, and CR was maintained up to 53.2 months. No transformation to DLBCL was observed in any patients during follow‐up.

### 3.5 Secondary treatment for non‐responders and relapsed cases

Among the 97 patients in the efficacy analysis set, 10 (10.3%) were transferred to secondary treatments. These 10 patients included 3 of the 4 relapsed patients who had initially achieved CR, and 7 of the 13 patients who did not achieve CR after successful *H. pylori* eradication. Secondary treatments included radiotherapy in 7 patients, rituximab monotherapy in 1 patient, radiotherapy combined with rituximab in 1 patient, and surgical resection in 1 patient with concomitant gastrointestinal stromal tumor of the stomach. Six of the 13 patients who did not achieve CR after successful *H. pylori* eradication therapy were followed up under a watch‐and‐wait strategy. The median interval to secondary treatment of MALT lymphoma was 12.3 months (range, 2.8‐29.3 months). Kaplan‐Meier estimates for the cumulative probability of secondary treatment transfer were 5.4% (95%CI, 2.3%‐12.6%) at 12 months, 10.1% (95%CI, 5.4%‐18.5%) at 24 months, and 11.5% (95%CI, 6.3%‐20.4%) at 36 months and at 48 months. The median interval to CR after secondary treatment was 2.1 months (range, 1.5‐12.9 months). Seventeen patients had shown treatment failures (4 patients relapsed after CR, 13 patients were non‐CR) (Figure 1).

### 3.6 Overall survival

During follow‐up, 3 of the 97 patients (3.1%) died of causes unrelated to MALT lymphoma. The OS rate was thus 96.9% (94/97). Median duration of follow‐up was 37.4 months (range, 2.4‐58.5 months), and 55.7% (54/97) of the efficacy analysis set were followed for more than 3 years. Kaplan‐Meier estimates for the cumulative probability of OS were 100% at 12 months, 98.8% (95%CI, 91.9%‐99.8%) at 24 months, and 97.2% (95%CI, 89.2%‐99.3%) at 36 months and at 48 months.

### 3.7 Other cancers

Other cancers detected after the start of eradication therapy were observed in 5 of 97 patients (5.2%), comprising 2 gastric cancers, 1 brain tumor, 1 esophageal cancer, and 1 pancreatic cancer. The interval from start of successful eradication treatment until detection of other cancer was 1.9 months and 5.9 months for the two gastric cancers, 10.2 months for the brain tumor, 27.5 months for the pancreatic cancer, and 41.5 months for the esophageal cancer.

### 3.8 Safety

Adverse reactions for *H. pylori* eradication were recorded in 3 of 107 patients (2.8%), comprising rash in 1 case, drug eruption in 1 case, and diarrhea and dysgeusia in 1 case. Apart from drug eruption, these reactions resolved spontaneously after the completion of eradication therapy. Only 1 patient developed a serious adverse reaction, the drug eruption, who was needed intravenous administration of prednisolone, and the reaction was attributed to penicillin allergy.

## 4 DISCUSSION

This was the first prospective, large‐scale, multicenter study under the regimens approved by Japanese Government for *H. pylori*‐positive gastric MALT lymphoma in Japan. In recent years, several large‐scale studies have investigated the efficacy of *H. pylori* eradication therapy against gastric MALT lymphoma. Among previous studies with around 100 subjects, CR was achieved in 80% (96/120) in a German multicenter prospective study,^17^ 94% (85/90) in a Korean prospective study,^18^ 85% (84/99) in a Korean single‐center prospective study,^19^ 74% (146/196) in a German single‐center retrospective study,^20^ 76% (78/102) in a joint Swiss‐Italian retrospective study,^21^ 77% (323/420) in a Japanese retrospective study,^15^ and 83% (78/94) in a Portuguese single‐center retrospective study.^22^ However, CR criteria varied among those studies. In a systematic review of 32 reports including those listed above,^23^ CR was found to have been achieved in 77% (1091/1408). The present study showed a high CR rate of 86.6% using *H. pylori* eradication therapy alone. As this is a prospective, nationwide, practice‐based, multicenter study, the CR rate might be reflecting a real‐world practice‐based result.

Previous studies have identified several factors associated with eradication resistance, including t(11;18)(q21;q21)/*API2–MALT1* translocation, absence of *H. pylori* infection, advanced clinical stage, deep submucosal invasion, and proximal localization in the stomach.^15^, ^23^, ^24^, ^25^ In this study, only 1 patient was positive for t(11;18)(q21;q21)/*API2–MALT1* translocation, all patients were *H. pylori*‐positive, 98% (95/97) showed stage I MALT lymphoma, and 10% (10/97) exhibited invasion beneath the submucosa. Therefore, we could not statistically clarify whether these factors correlated significantly with the efficacy of eradication treatment in this study, due to the small numbers of patients showing those factors.

Although the CR rate in this study was excellent, a significant factor related to CR rate was the type of endoscopic findings for gastric MALT lymphoma. Superficial type was the most common, appearing in 89 patients, and predicted a higher CR rate of 88.8% (79/89; 95%CI, 80.3%‐94.5%).

Tables 2 and 3 summarized 17 published studies that evaluated the efficacy of *H. pylori* eradication therapy in more than around 50 patients with *H. pylori*‐positive gastric MALT lymphoma. Table 2 indicates 9 retrospective studies 12, 15, 20, 21, 22, 26, 27, 28, 29 of 49‐376 patients. In total, CR was achieved in 852 of 1,055 patients (80.8%) including patients with successful and unsuccessful *H. pylori* eradication. CR rates in patients with stage I and stage II_1_ disease were 81.6% (823/1009) and 45.2% (33/73), respectively. Relapse of gastric MALT lymphoma was recorded in 64 of the 852 patients who achieved CR (7.5%), and treatment failure (relapse, PD, and/or non‐CR at ≥6 months after successful *H. pylori* eradication) was observed in 144 of all 1055 patients (13.6%). Table 3 summarizes 8 prospective studies,^17^, ^18^, ^19^, ^30^, ^31^, ^32^, ^33^ including the present investigation, of 47‐120 patients. The present study (n = 97) represents the third largest among these prospective studies. Overall CR rates were 85.4% (579/678) in all treated patients. Rates of relapse and treatment failure were 4.3% (25/579) and 14.5% (98/678), respectively. The combined CR rate in the 8 prospective studies (85.4%) was significantly higher than in above 9 retrospective studies (80.8%, Table 2) (*P *=* *.014, Fisher's exact probability test). Although the influence of other factors affecting CR rate besides clinical stage cannot be excluded in each study, this statistical difference might be meaning in the evaluation of *H. pylori* eradication treatment to gastric MALT lymphoma. As the influence of follow‐up bias is likely to observe in retrospective studies (dropout in regression cases during follow‐up), the exact CR rate of gastric MALT lymphoma by *H. pylori* eradication treatment should be evaluated by the accumulation of prospective studies.

**Table 2:** Review of the literature on efficacy of *Helicobacter pylori* eradication for *H. pylori*‐positive gastric MALT lymphoma: Retrospective studies

Author (year)^Ref.^	All patients		Stage I		Stage II_1_		Median follow‐up (months)	Relapse (%)	Treatment failure^a^ (%)
	No .	CR cases (%)	No.	CR cases (%)	No.	CR cases (%)			
Pinotti (1997)^26^	49	30 (61)	49	30 (61)	0	‐	22	2 (6.6)	5 (10)
Savio (2000)^27^	76	71 (93)	75	71 (95)	1	0	28	6 (8.5)	6 (7.9)
Wündisch (2006)^20^	196	146 (75)	193	146 (76)	0	‐	27	5 (3.4)	18 (9.2)
Nakamura (2008)^28^	70	55 (79)	65	54 (83)	5	1 (20)	46	1 (1.8)	10 (14)
Stathis (2009)^21^	85	66 (78)	80	66 (83)	5	0	76	16 (24)	23 (27)
Andriani (2009)^29^	60	42 (70)	44	37 (84)	9	5 (56)	65	13 (31)	16 (27)
Ono (2010)^12^	50	48 (96)	43	42 (98)	7	6 (86)	76	0	2 (4.0)
Nakamura (2012)^15^	376	317 (84)	378	304 (80)	35	17 (49)	72	10 (3.2)	37 (9.8)
Moleiro (2016)^22^	93	77 (83)	82	73 (89)	11	4 (36)	109	11 (14)	27 (29)
Total	1055	852 (81)	1009	823 (82)	73	33 (45)	65	64 (7.5)	144 (14)

CR, complete remission.

a Relapse, progressive disease, and/or non‐CR at 6 months after eradication.

**Table 3:** Review of the literature on efficacy of *Helicobacter pylori* eradication for *H. pylori*‐positive gastric MALT lymphoma: Prospective studies

Author (year)^Ref.^	All patients		Stage I		Stage II_1_		Median follow‐up (months)	Relapse (%)	Treatment failure^a^ (%)
	No .	CR cases (%)	No.	CR cases (%)	No.	CR cases (%)			
Weston (1999)^30^	65	50 (77)	65	50 (77)	0	‐	23	0	2 (3.1)
Urakami (2000)^31^	47	42 (89)	47	42 (89)	0	‐	20	0	0
Fischbach (2004)^32^	90	73 (81)	90	73 (81)	0	‐	45	4 (5.4)	13 (14)
Wündisch (2005)^17^	120	96 (80)	120	96 (80)	0	‐	75	3 (3.1)	27 (23)
Hong (2006)^18^	90	85 (94)	76	72 (95)	14	13 (93)	45	8 (9.4)	13 (14)
Kim (2007)^19^	99	84 (85)	99	84 (85)	0	‐	41	5 (6.0)	20 (20)
Terai (2008)^33^	70	65 (93)	65	60 (92)	5	5 (100)	46	1 (1.5)	6 (8.6)
Sugizaki (present study)	97	84 (87)	95	83 (87)	2	1 (50)	37	4 (4.8)	17 (18)
Total	678	579 (85)	657	560 (85)	21	19 (90)	43	25 (4.3)	98 (14)

CR, complete remission.

a Relapse, progressive disease, and/or non‐CR at 6 months after eradication.

Because gastric MALT lymphoma shows a slow progression and an excellent prognosis generally, one option is to adopt a watch‐and‐wait approach using endoscopy and suitable histopathology, as long as no deterioration of any remaining lymphoma is recognized.^34^ The National Comprehensive Cancer Network guideline recommends adopting a watch‐and‐wait strategy for 6 months after successful eradication in symptom‐free cases, even if lymphoma remains.^6^ In the present study, 1 of the 4 patients relapsed after achieving CR and followed using a watch‐and‐wait strategy obtained a second CR during the follow‐up period, while 6 of 13 non‐CR patients were followed up using a watch‐and‐wait strategy, but did not achieved PD without secondary therapy. The duration of watch‐and‐wait before transfer to secondary treatment was >6 months in 80% (8/10) and ≥1 year in 50% (5/10) in this study.

Several limitations must be considered in the present study. First, as the timing of transfer to secondary treatment was judged by a physician himself, some patients might receive oncological therapy before possible achievement of CR by eradication, which might have resulted in a reduced final CR rate by *H. pylori* eradication. Second, central review of biopsy specimens was performed in only diagnostic confusing cases, which might have induced some heterogeneity in the histologic assessment. Third, the lack of t(11;18) assessment in 24 of the 97 patients (25%) might have affected the analysis of factors predicting the outcomes of eradication therapy.

In summary, the present prospective, nationwide, multicenter study from Japan demonstrated that *H. pylori* eradication therapy for localized *H. pylori*‐positive gastric MALT lymphoma had an 87% CR rate, 95% CR maintenance rate, a secondary treatment transfer rate of only 10%, and a 97% survival rate, and was associated with excellent prognosis in clinical practice. RPZ‐based triple therapy showed an *H. pylori* eradication rate of about 90% and was associated with only a small number of well‐known adverse reactions. We therefore concluded that RPZ‐based *H. pylori* eradication therapy is clinically useful and can be recommended as first‐line treatment for *H. pylori*‐positive localized gastric MALT lymphoma in Japan.

## CONFLICT OF INTEREST

Katsuya Sugizaki is an employee of Eisai Co., Ltd. Katsuya Sugizaki owns stocks and shares in Eisai Co., Ltd. Akira Tari has received research funding from Eisai Co., Ltd. and EA Pharma Co., Ltd. Toshiro Sugiyama has received research funding from Eisai Co., Ltd., EA Pharma Co., Ltd., Takeda Pharmaceutical Co., Ltd., Daiichi Sankyo Company, Ltd., and Otsuka Pharmaceutical Co., Ltd. Yasuhiko Kitadai, Ichiro Oda, Shotaro Nakamura, and Tadashi Yoshino have nothing to declare.

## **AUTHOR CONTRIBUTIONS**

*Guarantor of the article*: Toshiro Sugiyama

Katsuya Sugizaki, as the sponsor's (Eisai Co., Ltd.) employee in charge of this study, was involved in protocol planning, data interpretation, writing the first draft of the manuscript, and revising its final version. Akira Tari and Shotaro Nakamura were involved in protocol planning, patient recruitment, and data interpretation. Yasuhiko Kitadai and Ichiro Oda were involved in patient recruitment. Tadashi Yoshino was involved in protocol planning and data interpretation. Toshiro Sugiyama, as the principal investigator, had overall responsibility of the study and was involved in protocol planning, data interpretation, and revising final version of the manuscript. All authors approved the final version of the manuscript.
